# The Effect of Surrounding Greenness on Type 2 Diabetes Mellitus: A Nationwide Population-Based Cohort in Taiwan

**DOI:** 10.3390/ijerph18010267

**Published:** 2020-12-31

**Authors:** Hui-Ju Tsai, Chia-Ying Li, Wen-Chi Pan, Tsung-Chieh Yao, Huey-Jen Su, Chih-Da Wu, Yinq-Rong Chern, John D. Spengler

**Affiliations:** 1 Institute of Population Health Sciences, National Health Research Institutes, Maioli 35053, Taiwan; tsaihj@nhri.org.tw; 2 Department of Environmental and Occupational Health, National Cheng Kung University, Tainan 70101, Taiwan; l8011213@hotmail.com (C.-Y.L.); hjsu@mail.ncku.edu.tw (H.-J.S.); 3 Institute of Environmental and Occupational Health Sciences, National Yang-Ming University, Taipei 112304, Taiwan; wenchipan@ym.edu.tw; 4 Division of Allergy, Asthma, and Rheumatology, Department of Pediatrics, Chang Gung Memorial Hospital and Chang Gung University College of Medicine, Taoyuan 333323, Taiwan; yao@adm.cgmh.org.tw; 5 Chang Gung Immunology Consortium, Chang Gung Memorial Hospital and Chang Gung University College of Medicine, Taoyuan 333323, Taiwan; 6 Community Medicine Research Center, Chang Gung Memorial Hospital at Keelung, Keelung 20401, Taiwan; 7 Department of Geomatics, National Cheng Kung University, Tainan 70101, Taiwan; dcpast1208.rive@gmail.com; 8 National Institute of Environmental Health Sciences, National Health Research Institutes, Miaoli 35053, Taiwan; 9 Department of Environmental Health, Harvard T.H. Chan School of Public Health, Boston, MA 02115, USA; spengler@hsph.harvard.edu

**Keywords:** surrounding greenness, type 2 diabetes mellitus, normalized difference vegetation index (NDVI), cohort study

## Abstract

This study determines whether surrounding greenness is associated with the incidence of type 2 diabetes Mellitus (T2DM) in Taiwan. A retrospective cohort study determines the relationship between surrounding greenness and the incidence of T2DM during the study period of 2001–2012 using data from the National Health Insurance Research Database. The satellite-derived normalized difference vegetation index (NDVI) from the global MODIS database in the NASA Earth Observing System is used to assess greenness. Cox proportional hazard models are used to determine the relationship between exposure to surrounding greenness and the incidence of T2DM, with adjustment for potential confounders. A total of 429,504 subjects, including 40,479 subjects who developed T2DM, were identified during the study period. There is an inverse relationship between exposure to surrounding greenness and the incidence of T2DM after adjustment for individual-level covariates, comorbidities, and the region-level covariates (adjusted HR = 0.81, 95% CI: 0.79–0.82). For the general population of Taiwan, greater exposure to surrounding greenness is associated with a lower incidence of T2DM.

## 1. Introduction

Diabetes mellitus affected approximately 285 million adults around the world in 2010, of which 90% were type 2 diabetes mellitus (T2DM), with the greatest increases in developing countries [1,2,3]. In the past decades, an alarming rise in the incidence of T2DM has been observed in Asia [4,5]. T2DM and its associated complications, such as cardiovascular diseases, diabetic nephropathy, and diabetic retinopathy, result in a substantial medical and socioeconomic burden for patients, their families, and society [6,7].

A growing body of studies has documented the beneficial effects of surrounding greenness on human physical and mental health [8,9]. A previous study determined the effect of green space on survival after a stroke using a cohort of subjects with previous acute ischemic stroke, and showed a relationship between the proximity of the subject’s residence to green space and a greater survival rate after a stroke [10]. The World Health Organization conceptual framework of social determinants of health also indicates how economic, political, and social factors influence an individual’s socioeconomic status [11]. Green space may modify some social determinants which, in turn, play a role in determining health outcomes including T2DM [12]. Lifestyle intervention has also been demonstrated to reduce the risk of developing T2DM [13], but the effectiveness depends on the neighborhoods where people live [14]. On the other hand, Dudek and colleagues reported that exposure to natural communities, such as forests, may pose an increased risk of allergy [15]. To date, limited studies have been conducted to determine the relationship between surrounding greenness and the incidence of T2DM.

This retrospective cohort study uses nationwide, population-based medical claims data for Taiwan. The study determines whether there is a relationship between surrounding greenness and the incidence of T2DM. The joint modifiable effects of several T2DM-related factors on the effect of surrounding greenness on the incidence of T2DM are also evaluated.

## 2. Materials and Methods

### 2.1. Study Population and Data Sources

Taiwan has had a single-payer National Health Insurance (NHI) program since 1995. Approximately 99.9% of the total population of Taiwan was enrolled in the NHI program by the end of 2014. For research purposes, the Taiwanese National Health Insurance Research Database (NHIRD) uses the NHI program’s medical claims data, including comprehensive information about patients’ demographic characteristics, diagnoses, and prescription claims data, medical facilities, and medical services [16]. All data related to personal identification in NHIRD was scrambled before being released to researchers. This retrospective cohort study uses one of the NHIRD subsets, the Longitudinal Health Insurance Database 2000 (LHID2000), which contains all original claims data from 2000 to 2012. A total of 1,000,000 individuals were randomly sampled from the 2000 Registry for beneficiaries of the NHI program (roughly 5% of the total population of Taiwan). The study subjects resided in 350 townships on the island of Taiwan between 2001 and 2012. This study was approved by the Institutional Review Board of the National Health Research Institutes, Taiwan, and the National Cheng Kung University Governance Framework for Human Research Ethics (104-081). All methods were carried out in accordance with relevant guidelines and regulations.

### 2.2. Study Subjects

**Figure 1:**
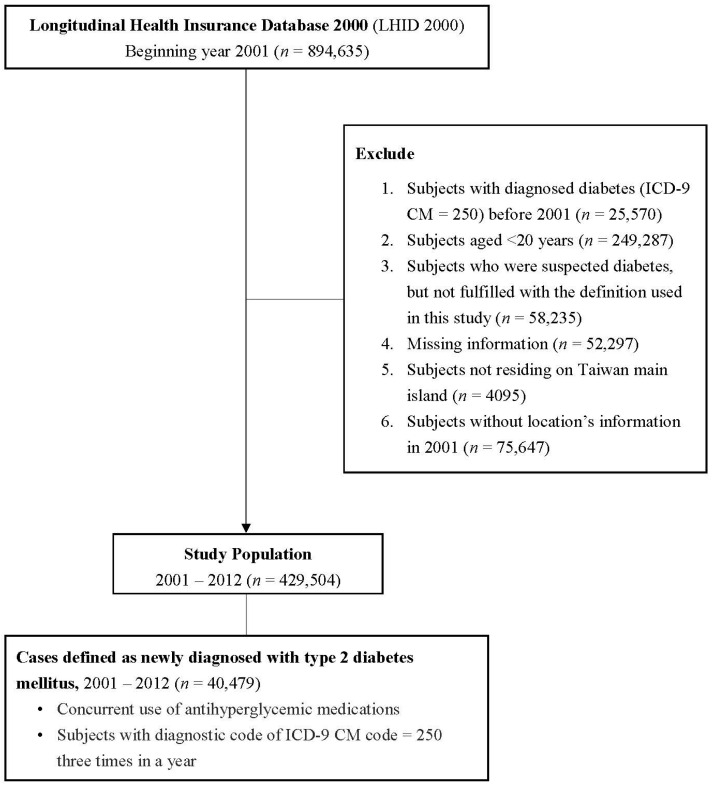
Flow chart for inclusion/exclusion criteria for the study subjects in 2001–2012.

The study subjects came from the LHID2000. The selection criteria are as follows: (1) Subjects aged 20 years or older in 2001; (2) subjects free of T2DM (based on International Classification of Diseases, Ninth Revision, Clinical Modification (ICD-9-CM) code: 250) before 1 January 2001; (3) subjects residing on the island of Taiwan; and (4) subjects registered as NHI beneficiaries in 2001–2012. The exclusion criteria are as follows: (1) Subjects aged less than 20 years in 2001; (2) subjects with T2DM diagnosis before 2001; and (3) subjects residing in Penghu, Kinmen, and Matsu Islands. A total of 429,504 subjects are included for subsequent analysis in this study. The detailed flowchart for inclusion/exclusion criteria for the study subjects is shown in Figure 1.

The study outcome is subjects who are diagnosed with T2DM. Subjects are determined to have T2DM if: (1) Subjects have three times or more ICD-9-CM code = 250 from inpatient or outpatient medical claims data within a year; and (2) subjects show concurrent use of anti-hyperglycemic medications [17,18].

### 2.3. Greenness Assessment

The surrounding greenness (the amount of trees and vegetation) for each subject is measured using a satellite-derived normalized difference vegetation index (NDVI). This is a spectrum-based greenness index that is defined as the ratio of absorbed visible light and reflected near-infrared to the total amount of visible and near infrared radiation striking a surface for measuring plant growth (vigor), vegetation cover, and biomass production from multispectral satellite data [19]. Global NDVI data come from the global Moderate Resolution Imaging Spectroradiometer (MODIS) database in the National Aeronautics and Space Administration (NASA) Earth Observing System [20,21]. Global MODIS NDVI Version 5 (NASA, Washington, D.C., USA) is updated every 16 days, at a spatial resolution of 250 m and the NDVI ranges from −1.0 to 1.0. A more positive NDVI value means a greater amount of healthy green vegetation [22]. In this study, all of the NDVI images from 2001 to 2012 are used for the subsequent analysis. Specifically, NDVI from 2001 to the event-month, death month, or end of follow-up was averaged to represent the long-term continuous time-varying exposure of greenness to subjects. In considering confidentiality issues, the residential address of subjects is not available in this study. The averaged exposure to greenness for each participant is determined using the location of the most frequently visited medical facilities each year as close proximity to greenness for study subjects. Circular buffers of 250 m, 500 m, and 1000 m, separately, were selected based on each medical facility to estimate subjects’ exposure to greenness within walking distance and a range of using common transportation [23].

### 2.4. Covariates

Potential confounding variables for this study include demographic information, such as age group (20–34, 35–44, 45–54, 55–64, and >65 years), gender (male or female), socioeconomic status (SES), such as insurance amount, occupational type (governmental employee and teachers, soldiers, service industries, agriculture workers, fishing and animal husbandry, non-governmental organizations, and others), and subjects’ comorbidities (hypertension [ICD-9-CM codes = 401–405] and hyperlipidemia [ICD-9-CM code = 272]). Contextual socioeconomic circumstances are controlled and adjusted, for example, by county-level income information (scaled from 0–625, 625–1250, 1250–1875 and >1875 in USD/year) from the Taiwanese National Statistics and township urbanization levels (scaled from level 1: Urban core area; level 2: Downtown; level 3: Emerging town; level 4: Traditional industry area; level 5: Low development town; level 6: Aging society town; level 7: Rural area).

### 2.5. Statistical Analysis

The distributions of baseline characteristics for the two NDVI exposure groups (using the four-quantile level of NDVI as the cut-off) are compared. An ANOVA-test are used for continuous variables, and a chi-square test is used for discreet variables. As the value of greenness was determined at the township level using the location of the most frequently visited medical facilities, Cox proportional hazard models were used with a generalized estimating equation to account for clustering for the same township, in order to determine the relationship between exposure to NDVI and the incidence of T2DM, with adjustment of any potential confounders [24]. The time at risk for T2DM is calculated for study subjects as the duration from 2001 (the enrollment year) to the date on which the patient is diagnosed with T2DM, death, or the last day of 2012 (the last observational year). The follow-up duration (using years as a unit) is the time variable in the models. The degree of exposure to greenness is calculated based on average greenness for each year until subjects develop the outcome, die, or follow-up ends.

Subgroup analyses are applied to determine the modifiable effects of various characteristics on the risk of developing T2DM risk, in terms of age group, sex, insurance amount, and comorbidity, including any diagnosis of hypertension or dyslipidemia. A sensitivity analysis is used to test robustness of the results. The analysis was then restricted to subjects who did not relocate during the study period, those who had a T2DM diagnosis every year, those who did not have relief aids or those who did not have coronary artery disease (CAD). Different buffer sizes (i.e., 250 m, 500 m, and 1000 m) were used to denote different degrees of exposure to greenness.

Statistical significance is determined using a 95% confidence interval (CI) or a *p*-value of less than 0.05. All of the analyses are performed using the statistical software, SAS 9.4 (SAS Institute, Cary, NC, USA) and R version 3.3.0 (The R project for statistical computing) (The R Foundation, Vienna, Austria).

## 3. Results

### 3.1. Baseline Characteristics for the Study Subjects

A total of 429,504 subjects were included in subsequent analyses. Of the eligible subjects, 40,479 developed T2DM during the study period (2001–2012). The mean follow-up time was 11.28 years, and the 12-year cumulative incidence is 9.4%. Table 1 shows the baseline characteristics for the study subjects, grouped in terms of high or low exposure to greenness, using a median level of NDVI (0.46) as the cut-off value. The distributions of age, sex, insurance amount, occupational types, township urbanization levels, county level income, and the incidence of T2DM were classified in terms of high or low exposure to greenness (Table 1).

### 3.2. The Relationship between Greenness and the Incidence of T2DM

Table 2 presents three different models involved in the adjustment of various covariates provided as follows: (1) The individual-level covariates; (2) the individual-level covariates and comorbidities; and (3) the individual-level covariates, comorbidities, and the region-level covariates. The results showed a significant inverse relationship between greenness (in terms of the per IQR increase) and the incidence of T2DM. There was a similar inverse relationship when greenness was modeled in quartiles.

### 3.3. Subgroup Analysis of Various Factors That Are Related to T2DM

Similar to the above Table 2, the results of the subgroup analyses in Table 3 showed that there was an inverse relationship between the surrounding greenness and the incidence of T2DM across all strata, for example, age, sex, insurance amount, hypertension, and hyperlipidemia, respectively.

### 3.4. Sensitivity Analysis

Sensitivity analyses using different criteria to determine the degree of exposure to greenness in subjects with different specific conditions indicated similar results (Table 4). First, for patients with no relocation, we intended to examine whether the results from main model were comparable to the subgroup exposed to stable degree of greenness, that is, no relocation. Second, for patients with diabetes diagnosis using anti-hyperglycemic medications every year, we intended to evaluate whether the results from main model were comparable to the subgroup with a relatively restricted diagnosis of T2DM. Third, for patients with no relief aids, we attempted to investigate whether the results from main model were comparable to the subgroup without considering low level of socio-economic status, specifically accounting for a crucial social determinant. Fourth, for patients with no coronary artery disease (CAD), we attempted to examine whether the results from main model were comparable to a relatively healthy subgroup. For subjects who experienced different conditions, an inverse relationship between exposure to greenness and the incidence of T2DM was found. The results in Table 4 also showed a similar inverse relationship with the incidence of T2DM for different sizes of buffer for surrounding greenness.

## 4. Discussion

This study suggests that a greater degree of surrounding greenness is associated with a reduced risk of T2DM. The inverse relationship between surrounding greenness and the incidence of T2DM is significant after adjusting for various individual-level risk factors, comorbid conditions and regional-level variables. Consistent inverse relationship was found when applying different criteria to determine the degree of exposure to surrounding greenness, or subjects with different specific conditions. To the best of the authors’ knowledge, this study is one of the first to determine the potential beneficial effect of exposure to surrounding greenness on the incidence of T2DM in a general population, particularly in an Asian country.

Previous studies have demonstrated that forest bathing trips affected human immune function, leading to an increase in natural killer (NK) activity via increasing the levels of intracellular granulysin and performance in and the number of NK cells [25,26,27]. Thus, it was likely exposure to surrounding greenness might decrease the risk of developing T2DM through modulating immune function. In addition, Cho et al. have reported that the beneficial effects of forest bathing on human health might be through terpenes, the largest class of naturally occurring organic compounds, which have the effect of anti-inflammatory, anti-cancer, or neuroprotective activities [28]. Higher exposure to surrounding greenness reducing the risk of developing T2DM may be partly due to higher levels of terpenes. Over the past years, growing evidence has suggested greenness was associated with health outcomes, such as obesity, mental health, and coronary heart disease [29] For example, Fong et al. have reported there were consistent results supporting the protective effects of greenness on various health outcomes, such as birth outcomes and mental health [30]. Our findings were in line with previous reports. In a US sample of 249,405 Medicare subjects aged 65 years or more, greenness might reduce various chronic conditions, including diabetes, hypertension, and hyperlipidemia [31]. In a UK study of 6076 participants, greater levels of greenness decreased the risk of metabolic syndrome (e.g., reduced levels of fasting glucose and/or triglyceride) [32]. In a sample of Australians aged 45 years and older, proximity to green space was associated with reduced body weight [33]. In an observational study using a large population-based Canadian birth cohort, Perry and colleagues reported that increased levels of residential greenness were associated with positive birth outcomes [34]. In a Dutch population, Maas and colleagues demonstrated there was an inverse relationship between proximity to green space and the incidence of T2DM, similar to the findings reported by Astell-Burt and colleagues in a group of 267,072 Australians aged 45 years and older [35,36]. Similarly, our results for this study showed that higher exposure to greenness was associated with a reduced risk of T2DM.

The mechanisms dictating the association between surrounding greenness and the incidence of T2DM are largely unclear. Previous reports have suggested that surrounding greenness may be associated with less exposure to ambient air pollution [29,37]. Proximity to surrounding greenness has also been reported to be associated with an increase in physical activity, which is positively associated with a healthy lifestyle [38]. Roe et al. have noted that changes in salivary cortisol, which is a measure of stress, are related to exposure to greenness, and as a result, may affect susceptibility to T2DM [39].

To the best of the authors’ knowledge, this is the first 12-year nationwide, population-based study to demonstrate the beneficial effect of different sizes of buffer (i.e., 250 m, 500 m, 1000 m, and 2000 m) of surrounding greenness on the incidence of T2DM in a general Asian population in Taiwan. This inverse relationship between exposure to surrounding greenness and T2DM exists, regardless of whether the model is adjusted for individual-level covariates, such as age, sex, insurance amount, and occupation types, or for region-level covariates, such as county-level income information and township urbanization levels.

On the other hand, some limitations should be noted. First, information about potential confounding factors, such as diet, smoking, alcohol, and obesity, are not available in the NHIRD. However, we carried out sensitivity analyses. Similar results were observed when restricting subjects without CAD, which may be partially controlled for obesity and/or low physical activity [16,17]. Still, it was likely that there were residual confounding effects due to unmeasured confounders. Second, due to the lack of residential location information, the individuals’ exposure data could only be estimated based on the location of the most frequently visited medical centers. It might cause potential misclassification of exposure estimation and not be fully representative of the individuals’ exposure. Third, information of the types of green spaces, their sizes, and the distances are not available in this study. We are not able to examine their influence on the incidence of T2DM. Thus, the results should be interpreted with caution. The present study did not show the dose-response relationship between greenness and T2DM. However, we observed that the increasing greenness exposure reduced the risk of T2DM in the second, third, and fourth quartile for greenness. Further evaluation of potential dose-response relationship will be needed to clarify whether such relationship exits. The effect of surrounding greenness on the incidence of T2DM is determined for a Taiwanese cohort. The results for this study may or may not be generally applicable to other populations.

## 5. Conclusions

The results of this study show that there is an inverse relationship between surrounding greenness and the incidence of T2DM. The results remained consistent when different criteria were used to define the degree of exposure to surrounding greenness, or for subjects with different specific conditions. Further study is required to confirm the results of this study. It is of importance to determine the underlying mechanisms by which surrounding greenness affects the incidence of T2DM.

## Figures and Tables

**Table 1:** Baseline characteristics for study subjects (*n* = 429,504), grouped in terms of the quantile for normalized difference vegetation index (NDVI) in 2001.

Variables at Baseline	NDVI < Q1 (0.389)	Q1 (0.389) ≤ NDVI < Q2 (0.478)	Q2 (0.478) ≤ NDVI < Q3 (0.534)	Q3 (0.534) ≤ NDVI	*p* Value *
	(*n* = 105,589)	(*n* = 108,909)	(*n* = 112,899)	(*n* = 102,107)	
Age (years) (mean ± SD) ^a^	42.85 ± 16.35	41.58 ± 15.90	40.76 ± 15.59	42.09 ± 16.44	<0.01
Age, *n* (%)					
20–34	39,111 (36.86%)	43,560 (39.35%)	42,370 (41.13%)	42,767 (38.99%)	<0.01
35–44	24,066 (22.68%)	26,750 (24.16%)	24,496 (23.78%)	25,403 (23.16%)	
45–54	18,867 (17.78%)	18,518 (16.73%)	17,448 (16.94%)	17,948 (16.36%)	
55–64	10,677 (10.06%)	9688 (8.75%)	8757 (8.50%)	10,035 (9.15%)	
65+	13,381 (12.61%)	12,194 (11.01%)	9943 (9.65%)	13,525 (12.33%)	
Sex, *n* (%)					
Male	51,455 (48.50%)	53,012 (47.88%)	49,202 (47.76%)	53,655 (48.92%)	<0.01
Female	54,647 (51.50%)	57,698 (52.12%)	53,812 (52.24%)	56,023 (51.08%)	
National Health Insurance premium (mean ± SD)	611.45 ± 538.11	606.59 ± 510.40	604.16 ± 523.60	594.48 ± 495.96	<0.01
*n* (%)					
USD$0–645.2	69,329 (65.34%)	74,021 (66.86%)	68,243 (66.25%)	75,426 (68.77%)	<0.01
USD$645.2–1290.3	23,571 (22.22%)	24,595 (22.22%)	22,903 (22.23%)	23,576 (21.50%)	
USD$1290.3–1935.5	13,137 (12.38%)	12,034 (10.87%)	11,773 (11.43%)	10,591 (9.66%)	
USD$1935.5+	65 (0.06%)	60 (0.05%)	95 (0.09%)	85 (0.08%)	
Occupational type, *n* (%)					
Official staff and teacher	11,995 (11.31%)	10,355 (9.35%)	9730 (9.45%)	10,620 (9.68%)	<0.01
Soldier	894 (0.84%)	885 (0.80%)	1076 (1.04%)	1050 (0.96%)	
Services-producing industries	65,699 (61.92%)	68,479 (61.85%)	66,792 (64.84%)	65,827 (60.02%)	
Agriculture, forestry, fishing, and animal husbandry	10,504 (9.90%)	15,302 (13.82%)	10,651 (10.34%)	16,703 (15.23%)	
Nonprofit organization	2216 (2.09%)	1831 (1.65%)	1620 (1.57%)	1524 (1.39%)	
Relief aids	14,473 (13.64%)	13,624 (12.31%)	12,880 (12.50%)	13,640 (12.44%)	
Others	321 (0.30%)	234 (0.21%)	265 (0.26%)	314 (0.29%)	
Township urbanization level, *n* (%)					
Urban core space (level 1)	35,353 (33.32%)	17,703 (15.99%)	35,801 (34.75%)	34,727 (31.66%)	<0.01
Downtown (level 2)	48,713 (45.91%)	48,013 (43.37%)	19,505 (18.93%)	27,778 (25.33%)	
Emerging town (level 3)	19,987 (18.84%)	34,174 (30.87%)	38,920 (37.78%)	17,825 (16.25%)	
Traditional industry town (level 4)	567 (0.53%)	7499 (6.77%)	5036 (4.89%)	8908 (8.12%)	
Low development town (level 5)	949 (0.89%)	3110 (2.81%)	3752 (3.64%)	19,464 (17.75%)	
Aging society town (level 6)	533 (0.50%)	0 (0.00%)	0 (0.00%)	976 (0.89%)	
Rural area (level 7)	0 (0.00%)	211 (0.19%)	0 (0.00%)	0 (0.00%)	
County level income (mean ± SD)	2714.45 ± 1516.36	2268.85 ± 1369.57	2521.32 ± 1398.44	1960.04 ± 1424.09	<0.01
Incident cases of type 2 diabetes, *n* (%)	13,186 (12.43%)	9579 (8.65%)	8061 (7.83%)	9653 (8.80%)	<0.01

* Compare the median by using Wilcoxon rank-sum test for age, national health insurance premium, and county level income, Pearson’s chi-squared test for age group, insurance amount group, occupational type, and township urbanization level, and Fisher’s exact test for sex and type 2 diabetes status. ^a^ Standard Deviation.

**Table 2:** Association between long-term greenness exposure and incidence of type 2 diabetes mellitus.

Model	No. of Participants (Cases)	HR (95% CI) ^†^	HR (95% CI) ^‡^	HR (95% CI) ^§^	HR (95% CI) ^||^
Quartiles of NDVI					
First quartile (NDVI ≤ 0.39)	107,782 (13,667)	ref	ref	ref	ref
Second quartile (0.39 < NDVI ≤ 0.46)	109,030 (9098)	0.64 (0.62–0.66)	0.70 (0.68–0.72)	0.73 (0.71–0.75)	0.71 (0.69–0.73)
Third quartile (0.46 < NDVI ≤ 0.51)	103,014 (8061)	0.60 (0.58–0.62)	0.68 (0.66–0.70)	0.70 (0.68–0.72)	0.68 (0.66–0.70)
Forth quartile (0.51 < NDVI ≤ 0.78)	109,678 (9653)	0.68 (0.66–0.70)	0.71 (0.69–0.73)	0.72 (0.70–0.74)	0.72 (0.70–0.75)
Per IQR * increment	429,504 (40,479)	0.79 (0.78–0.80)	0.80 (0.80–0.81)	0.83 (0.82–0.84)	0.80 (0.71–0.90)

HR, hazard ratio; CI, confidence interval; IQR, interquartile range. * IQR = 0.11465. ^†^ Crude model. ^‡^ Adjusted for individual-level covariates: Age group, sex, level of insurance amount, and occupational type. ^§^ Adjusted for individual-level covariates and two comorbid conditions (hypertension and hyperlipidemia). ^||^ Adjusted for individual-level covariates, two comorbid conditions, and regional covariates (air pollution, county level income, and township level urbanization).

**Table 3:** Association between greenness exposure and incidence of type 2 diabetes mellitus, stratified by age, sex, insurance amount, hypertension, and hyperlipidemia, respectively.

Characteristics	No. of Participant (Case)	HR (95% CI) *
Age (year) ^†^		
<65	380,461 (28,061)	0.77 (0.76–0.78)
≥65	59,043 (12,418)	0.87 (0.84–0.89)
Sex ^‡^		
Male	207,324 (22,132)	0.81 (0.79–0.82)
Female	222,180 (18,347)	0.81 (0.79–0.82)
Insurance amount (USD$) ^§^		
Low	191,814 (16,359)	0.79 (0.77–0.81)
High	237,690 (24,120)	0.80 (0.79–0.82)
Income (County) (USD$) ^#^		
Low	241,378 (23,686)	0.80 (0.79–0.82)
High	188,126 (16,793)	0.78 (0.76–0.79)
Township Urbanization Level ^δ^		
Low urbanization	161,911 (15,880)	0.89 (0.86–0.91)
High urbanization	267,593 (24,599)	0.78 (0.76–0.79)
Hypertension ^η^		
No	314,089 (16,517)	0.80 (0.78–0.81)
Yes	115,415 (23,962)	0.82 (0.80–0.84)
Hyperlipidemia ^η^		
No	356,508 (24,700)	0.80 (0.79–0.82)
Yes	72,996 (15,779)	0.80 (0.78–0.82)

* IQR = 0.11465. ^†^ Model was adjusted for sex, level of insurance amount, occupational type, hypertension, hyperlipidemia, county level income, and township urbanization. ^‡^ Model was adjusted for age group, level of insurance amount, occupational type, hypertension, hyperlipidemia, county level income, and township urbanization. ^§^ Model used the median amount of the insurance (USD$600) as the cutoff value and adjusted for age group, sex, occupational type, hypertension, hyperlipidemia, county level income, and township urbanization. ^#^ Model used the median amount of the county level income (USD$2280.28) as the cutoff value and adjusted for age group, sex, level of insurance amount, occupational type, hypertension, hyperlipidemia, and township urbanization. ^δ^ Model stratified by township urbanization level 1–2, 3–7, and adjusted for age group, sex, level of insurance amount, occupational type, hypertension, hyperlipidemia, and county level income. ^η^ Model was adjusted for age group, sex, level of insurance amount, occupational type, Hypertension (or Hyperlipidemia), county level income, and township urbanization.

**Table 4:** The relationship between long-term exposure to greenness and the incidence of type 2 diabetes mellitus.

Model *	No. of Subjects (Cases)	HR (95% CI)
**Restricted to subjects with the following conditions**		
No relocation	226,192 (23,634)	0.83 (0.82–0.85)
Diabetes diagnosis with using anti-hyperglycemic medications every year	412,563 (23,538)	0.80 (0.78–0.81)
No relief aids	374,887 (34,635)	0.81 (0.80–0.82)
No CAD	355,509 (23,966)	0.76 (0.67–0.87)
**Using different buffer sizes**		
Buffer 250 m	429,504 (40,479)	0.81 (0.79–0.82)
Buffer 500 m	429,504 (40,479)	0.94 (0.93–0.96)
Buffer 1000 m	429,504 (40,479)	0.94 (0.92–0.95)

HR, hazard ratio; CI, confidence interval; CAD, coronary artery disease. * Model is adjusted for age group, sex, level of National Health Insurance premium, occupational type, hypertension, hyperlipidemia, county level income, and township urbanization.

## Data Availability

No new data were created or analyzed in this study. Data sharing is not applicable to this article.
